# Dual immunomodulatory effects of *bacteroides and phocaeicola* secretomes revealed by integrative metabolomic and functional screening in HT-29 cells

**DOI:** 10.3389/fmicb.2026.1897419

**Published:** 2026-08-11

**Authors:** Irina V. Podoprigora, Natalya B. Zakharzhevskaya, Milana S. Das, Andrei V. Chaplin, Dmitry A. Kardonsky, Elizaveta A. Vorobyeva, Daria A. Kashatnikova, Victoriia D. Kazakova, Irina V. Kolesnikova, Svetlana V. Smirnova, Tatiana N. Kalachnuk, Razan Marouf, Boris A. Efimov

**Affiliations:** 1 Department of Microbiology named after V.S. Kiktenko, Peoples’ Friendship University of Russia named after Patrice Lumumba (RUDN University), Moscow, Russia; 2 Department of Microbiology and Virology, Institute of Preventive Medicine named after Z.P. Solovyov, Pirogov Russian National Research Medical University, Moscow, Russia; 3 Lopukhin Federal Research and Clinical Center of Physical-Chemical Medicine of Federal Medical Biological Agency, Moscow, Russia; 4 Research Institute of Molecular and Cellular Medicine, Peoples’ Friendship University of Russia Named after Patrice Lumumba (RUDN University), Moscow, Russia; 5 The Laboratory of Ecological Genetics, Vavilov Institute of General Genetics, Russian Academy of Sciences, Moscow, Russia

**Keywords:** *Bacteroides*, conditioned medium, IL-8 secretion, inflammation, intestinal homeostasis, metabolites, *Phocaeicola*, TLR2/TLR4 gene expression

## Abstract

**Background:**

The immunomodulatory effects of gut *Bacteroides* are typically attributed to surface polysaccharides signaling through TLR2 and TLR4, and to outer membrane vesicles (OMVs) that traffic effector molecules to host cells. However, these bacteria also secrete a wide array of metabolites, including short-chain fatty acids (SCFAs), medium-chain fatty acids (MCFAs), phenolic compounds, and aldehydes, whose contributions to immune modulation remain underexplored. Moreover, it is unclear whether different *Bacteroides* and *Phocaeicola* species share similar immunomodulatory profiles or display species- and strain-specific activities.

**Methods:**

We cultivated 18 strains of *Bacteroides* and *Phocaeicola*, collected their conditioned media, and applied them to HT-29 intestinal epithelial cells. *TLR2* and *TLR4* gene expression and IL-8 secretion were measured under basal and LPS-stimulated conditions. The volatile metabolome was characterized using HS-GC/MS, and the data were integrated with cellular responses.

**Results:**

The strains induced strikingly divergent effects, ranging from strong suppression to marked induction of *TLR2/TLR4* expression and IL-8 production. Metabolomic profiling revealed that while SCFAs and MCFAs were associated with anti-inflammatory outcomes, the presence of phenolic compounds and aldehydes correlated with pro-inflammatory effects. Integration of metabolomic and cellular data allowed us to classify the strains into three functional categories: anti-inflammatory (e.g., *B. uniformis* EBA 5-20), pro-inflammatory (e.g., *B. thetaiotaomicron* 6-237), and strains with context-dependent mixed effects.

**Conclusion:**

The immunomodulatory activity of gut *Bacteroides and Phocaeicola* cannot be predicted solely by their taxonomic affiliation; rather, it depends on the specific repertoire of secreted metabolites and their interplay with host pathways. Evaluating both metabolomic profiles and receptor-mediated responses provides a rational basis for selecting candidate probiotics for personalized microbiome modulation.

## 1 Introduction

The taxonomic diversity of the intestinal microbiome is mainly composed of bacteria belonging to six dominant phyla: *Bacteroidota*, *Bacillota*, *Pseudomonadota*, *Actinobacteriota*, *Verrucomicrobia*, and *Fusobacteriota*. Bacteria from the phyla *Bacteroidota* and *Bacillota* comprise up to 90% of the human gut microbiota, and the balance between them is considered an important, though not absolute, indicator of health (Petersen and Round, 2014; Trosvik and de Muinck, 2015; Stojanov et al., 2020). The phylum *Bacteroidota* includes numerous representatives of the order *Bacteroidales*, most of which belong to the families *Bacteroidaceae*, *Prevotellaceae*, *Rikenellaceae*, *Barnesiellaceae*, *Odoribacteraceae*, and *Tannerellaceae* (Rajilić-Stojanović and De Vos, 2014). Intestinal bacteria belonging to the family *Bacteroidaceae*, which typically dominates among closely related taxa, are strictly anaerobic, non-spore-forming, Gram-negative rods whose abundance may exceed 10^10^ colony-forming units per gram (CFU/g) of feces (Efimov et al., 2019). Many bacterial species of the genera *Bacteroides* and *Phocaeicola* are highly adapted to the intestinal environment (Wexler, 2007; Shin et al., 2024). For example, *Bacteroides fragilis* is capable of growing under conditions of incomplete anaerobiosis in the presence of low concentrations of molecular oxygen (Baughn and Malamy, 2004).

Possessing a wide range of microbe-associated molecular patterns (MAMPs) on their surface, *Bacteroides* species can influence the effector mechanisms of innate immunity through pattern recognition receptors (PRRs), including Toll-like receptors (TLRs), NOD-like receptors (NLRs), and C-type lectin receptors (CLRs). Activation of these receptors induces the secretion of cytokines and antimicrobial peptides by epithelial cells and intraepithelial leukocytes. Stappers et al. (2012) demonstrated that TLR2, TLR1, and NOD2 receptors expressed on human peripheral blood mononuclear cells mediate recognition of *B. fragilis* outer membrane MAMPs, resulting in strong stimulation of innate immunity and pronounced induction of IL-6 and IL-8 secretion, which may explain the ability of *B. fragilis* to cause intra-abdominal abscesses. At the same time, the capsular polysaccharides A and B (PS-A and PS-B) of *B. fragilis*, after uptake by antigen-presenting cells and subsequent oxidative processing into smaller fragments, can bind to MHC class II molecules and be presented to T lymphocytes. This interaction plays an important role in CD4^+^ T cell activation, leading to the production of IL-10, which prevents abscess formation and other inflammatory reactions. Although polysaccharides are typically considered B-cell activators that stimulate increased IgM production, this mechanism illustrates their broader immunomodulatory potential (Mazmanian and Kasper, 2006; Zhou et al., 2022).

In addition, members of the genus *Bacteroides* are known to secrete outer membrane vesicles (OMVs) containing a variety of biologically active components, including proteins, DNA, RNA, lipopolysaccharides (LPS), and peptidoglycan (Zakharzhevskaya et al., 2017). Importantly, capsular polysaccharide A (PSA) is also associated with these vesicles. In a chemically induced colitis model in mice, administration of PSA-containing OMVs resulted in a significant improvement in clinical condition and a reduction in inflammatory responses, highlighting their potential role in immune regulation (Shagaleeva et al., 2024). Bacterial vesicles exert potent regulatory effects on the innate immune system by engaging TLR2, TLR4, TLR7, and NOD1-like receptors at a distance, whereas intact bacterial cells predominantly trigger activation of TLR2 (Gilmore et al., 2022). The same study demonstrated that OMVs are capable of penetrating human intestinal epithelial cells (Caco-2), suggesting their potential role in intercellular communication and the delivery of immunologically active components into host cells (Podoprigora et al., 2024).

Bacteria, including members of the genus *Bacteroides*, secrete a substantial array of metabolites that can contribute to the modulation of immune responses. The roles of short-chain fatty acids, medium-chain fatty acids, phenolic compounds, and aldehydes are particularly well characterized (Hays et al., 2024).

*Bacteroides* species ferment non-digestible carbohydrates, dietary fiber, and other polysaccharides to produce short-chain fatty acids (SCFAs), primarily acetate, propionate, and succinate (Liu et al., 2024). These metabolites are not only end products of fermentation but also serve as key carbon and energy sources for other members of the gut microbiota, including butyrate-producing bacteria such as *Faecalibacterium prausnitzii*, thereby sustaining the stability and functionality of the microbial consortium (Murakami et al., 2021). Beyond supporting commensal bacteria, SCFAs also contribute to host defense; for instance, elevated concentrations of acetate or butyrate can inhibit the growth of pathogenic bacteria by creating a low-pH environment (Zhang et al., 2020; Salvi and Cowles, 2021).

At the host level, SCFAs act as a critical energy source for epithelial cells, with butyrate in particular serving as the primary fuel for colonic epithelial cells, promoting their proliferation and supporting the integrity of the mucosal barrier (Schreiber et al., 2024). SCFAs also enhance barrier function by upregulating the expression of tight junction proteins, such as claudins and occludins, thereby reducing epithelial permeability and protecting against pathogen translocation (Schreiber et al., 2024). Furthermore, SCFAs interact with G-protein-coupled receptors (GPR41, GPR43, and GPR109A) expressed on immune and epithelial cells, stimulating the production of anti-inflammatory cytokines (e.g., IL-10) while suppressing pro-inflammatory mediators (IL-6, IL-8, TNF-*α*) (Du et al., 2024; Ranjbar et al., 2021). In addition, butyrate functions as a histone deacetylase (HDAC) inhibitor, modulating the epigenetic regulation of genes involved in immune responses (Liu et al., 2018). For *Bacteroides* species, both the extracellular spectrum of metabolites they release into the environment and the volatile metabolites packaged within their vesicles have been characterized. These vesicle-associated metabolites can be taken up by epithelial cells and influence intracellular signaling pathways, thereby modulating immune responses (Shagaleeva et al., 2023; Sheikh et al., 2025).

Taken together, these studies highlight the complexity of interactions between *Bacteroides* species and the human immune system. However, the modulation of cellular and immune activity can vary even within a single genus. Specifically, individual *Bacteroides* species differ in their enzymatic activity, the level of secretion of various components, and potentially in the mechanisms of their interaction with cell-surface receptors. Given the importance of these bacterial taxa in maintaining human health or contributing to disease development, the aim of the present study was to investigate the immunomodulatory properties of 18 strains representing 16 bacterial species of the genera *Bacteroides* and *Phocaeicola* within the family *Bacteroidaceae*: *B. uniformis*, *B. thetaiotaomicron*, *B. caccae*, *B. eggerthii*, *B. stercoris*, *B. intestinalis*, *B. clarus*, *B. salyersiae*, *B. xylanisolvens*, *B. finegoldii*, *B. cellulosilyticus*, *B. fragilis*, *P. dorei*, *P. vulgatus*, *P. coprocola*, and *P. plebeius*. The *in vitro* model involved stimulation of the intestinal epithelial cell line HT-29 with pro-inflammatory stimuli, including bacterial cell wall components (*E. coli* LPS) and conditioned media containing metabolites and cell wall–associated molecular patterns produced by the tested strains of *Bacteroides* and *Phocaeicola*. By integrating metabolomic data with the assessed gene expression levels, we aimed to evaluate the overall immunomodulatory effect of the studied bacterial species, classifying them into groups with anti-inflammatory or pro-inflammatory properties.

## 2 Materials and methods

### 2.1 Strains of the genera *Bacteroides* and *Phocaeicola*

In this study, 18 strains from the family *Bacteroidaceae* were investigated, including 14 *Bacteroides* strains: *B. uniformis* EBA 5–20 (Buni_5_20), *B. thetaiotaomicron* 6–237 (Bthe_6–237), *B. caccae* EBA 6–24 (Bcac_6_24), *B. eggerthii* 91 (Begg_91), *B. stercoris* 5888 (Bste_5888), *B. intestinalis* 181 (Bint_181), *B. clarus* 606 (Bcla_606), *B. salyersiae* 2,697 (Bsal_2,697), *B. xylanisolvens* EBA 5–17 (Bxyl_5_17), *B. xylanisolvens* Pik (Bxyl_Pik), *B. finegoldii* EBA 6–28 (Bfin_6_28), *B. cellulosilyticus* 807 (Bcel_807), *B. fragilis* BOB25 (Bfra_Bob25), and *B. fragilis* JIM10 (Bfra_JIM10); and 4 *Phocaeicola* strains (formerly *Bacteroides* until 2020 (Oren and Garrity, 2020)): *P. dorei* EBA 7–24 (Pdor_7_24), *P. vulgatus* EBA 3–9 (Pvul_3_9), *P. coprocola* EBA 6–21 (Pcop_6_21), and *P. plebeius* 2436 (Pple_2436). These strains were previously isolated and deposited in the collections of the Department of Microbiology and Virology, Pirogov Russian National Research Medical University, and the Lopukhin Federal Research and Clinical Center of Physical–Chemical Medicine, Federal Medical and Biological Agency (Moscow, Russian Federation). The strains have been characterized in our earlier studies (Shagaleeva et al., 2023; Efimov et al., 2019; Shkoporov et al., 2008), and their taxonomic identity was reconfirmed prior to the present experiments by MALDI-TOF MS and/or 16S rRNA gene sequencing (data not shown).

### 2.2 Bacterial and HT-29 cell line cultivation

Lyophilized bacterial strains were stored at −80 °*C. prior* to experimentation, the strains were plated on Anaerobe Basal Agar (Oxoid, Basingstoke, United Kingdom) supplemented with 5% (v/v) sheep blood. Following incubation at 37 °C for 48 h under anaerobic conditions, the strains were subcultured in Schaedler Anaerobe Broth (Oxoid, Basingstoke, United Kingdom) for 24 h. The culture supernatant was obtained after cultivation in Schaedler Anaerobe Broth.

The human intestinal epithelial cell line HT-29 was kindly provided by Dr. I. V. Arutyunyan, PhD, Head of the Laboratory of Cell Technologies and Tissue Engineering, Research Institute of Molecular Cell Biology, RUDN University. Cells were cultured in high-glucose DMEM (HG-DMEM; Capricorn Scientific, United States) supplemented with 10% (v/v) fetal bovine serum (Capricorn Scientific, United States), penicillin (100 U/mL) and streptomycin (100 μg/mL), and 1% L-glutamine (Corning, United States).

### 2.3 Conditioned media preparation

*Bacteroides* and *Phocaeicola* (except *P. plebeius* 2,436) were cultured in Schaedler Anaerobe Broth (Oxoid, Basingstoke, United Kingdom) for 24 h under anaerobic conditions. The *P. plebeius* 2,436 strain was grown in Basal Broth supplemented with 2% (w/v) dextrose at 37 °C under anaerobic conditions for 48–72 h. Following cultivation, all broth cultures were centrifuged (40 min, 3500g ) and resuspended in an equal volume of high-glucose DMEM (4.5 g/L) supplemented with 20 mM HEPES (Capricorn Scientific, United States). After a second centrifugation under the same conditions and removal of the supernatant, the bacterial suspensions were adjusted to approximately 3.0 × 10^8^ CFU/mL (corresponding to 1.0 McFarland standard) using a densitometer (Den-1; BioSan, Latvia–United Kingdom) in DMEM with 20 mM HEPES. The suspensions were then incubated for 20 h at 37 °C under anaerobic conditions. After incubation, bacterial cells were removed by centrifugation, and the resulting cell-free conditioned media were sterilized by filtration through a 0.22 μm membrane filter (Merck Millipore, Germany) for subsequent use in experiments.

### 2.4 HT-29 cell treatment and gene expression analysis

HT-29 cells were incubated with 450 μL per well of conditioned media or negative control media for 30 min. Subsequently, LPS from *Escherichia coli* O55:B5 (Sigma- Aldrich, United States) was added at 10 μL per well to achieve a final concentration of 100 ng/mL, and the cells were incubated for an additional 4 h. The experiment was performed in triplicate. Total RNA was isolated using the QIAGEN RNeasy Plus kits (Qiagen, Germany) according to the manufacturer’s instructions. Reverse transcription was performed for 1 h at 37 °C and 10 min at 70 °C to stop the reaction according to the manufacturer’s instructions. Quantitative real-time PCR (qRT-PCR) was performed using the Bio-Rad CFX 96 Real-Time Detection System (Bio-Rad, United States). Oligonucleotide primers and thermocycling conditions for the amplification are shown in Table 1. qPCRmix-HS-SYBR (Evrogen, Russia) was used to prepare the reaction mixtures. The specificity of reaction products was confirmed by melting temperature analysis (from 70 °C to 95 °C in 0.5 °C/15 s increments). The mRNA level of *GAPDH* was used to normalize the expression ratio of genes of interest. All reactions were carried out three times, and the final analysis was based on the mean of the reactions. The relative quantification method of 2^−ΔΔСt^ was used in further calculation; comparison of fold changes was made by the Welch’s one-way ANOVA with Games-Howell multiple comparisons test (Livak and Schmittgen, 2001). The oligonucleotide primers were the following Table 1.

**Table 1:** The oligonucleotide primers and PCR conditions.

Gene	Primer sequences (5′–3′)	PCR protocol	Reference
*GAPDH*	F-CTTTGACGCTGGGGCTGGCATT	**ID** 95 °C 5 min, **D** 96 °C 20 s, **A** 56 °C 30 s, **E** 72 °C 20 s, **NC** 40.	Khokhlova et al., 2022
	R-TTGTGCTCTTGCTGGGGCTGGT		
*IL8*	F-CAAGGAAAACTGGGTGCAGA	**ID** 95 °C 5 min, **D** 94 °C 20 s, **A** 60 °C 30 s, **E** 72 °C 20 s, **NC** 40.	Khokhlova et al., 2012
	R-CCTACAACAGACCCACACAA		
*TLR2*	F: ATCCTCCAATCAGGCTTCTCT	**ID** 95 °C 5 min, **D** 96 °C 20 s, **A** 55 °C 30 s, **E** 72 °C 20 s, **NC** 40.	Ma et al., 2023
	R: GGACAGGTCAAGGCTTTTTACA		
*TLR4*	F-GGTGGAAGTTGAACGAATGG	**ID** 95 °C 5 min, **D** 96 °C 20 s, **A** 60 °C 30 s, **E** 72 °C 20 s, **NC** 40.	Ren et al., 2019
	R-CCAGCAAGAAGCATCAGGTG		

### 2.5 IL-8 enzyme-linked immunosorbent assay

Supernatants were collected following 20 h of incubation and stored at −80 °C until analysis. A quantitative ELISA was performed using commercial kits (Vector-Best, Russia) according to the manufacturer’s protocol. The optical density was measured immediately at 495 nm using an iMark microplate reader (Bio-Rad, United States).

### 2.6 Hs-GC/MS

The culture media were prepared by removing bacteria from the culture broth via centrifugation and filtration through a 0.22 μm pore filter, yielding a sterile cell-free medium, in addition to the conditioned media prepared as described above. 200 μL culture or conditioned media samples plus 100 μL water samples were placed into 10 mL screw-cap vials for a Shimadzu HS-20 headspace extractor (Shimadzu, Japan). All HS-GC/MS analyses were performed using three independent biological replicates, with two technical replicates analyzed for each biological replicate. 0.2 g of a mixture of salts (ammonium sulfate and potassium dihydrogen phosphate in a ratio of 4:1) was added to increase solution ionic strength. Headspace extractor settings used: oven temperature 80 °C, sample line temperature 220 °C, transfer line temperature 220 °C, equilibrating time 15 min, pressurizing time 2 min, load time 0.5 min, injection time 1 min, needle flush time 7 min. The vials were sealed and analyzed on a Shimadzu.

QP2010 Ultra GC/MS with a Shimadzu HS-20 headspace extractor (Shimadzu, Japan), a VF-WAX MS column with a length of 30 m, a diameter of 0.25 mm, and a phase thickness of 0.25 μm. Initial column temperature 80 °C, heating rate 20 °C/min to 240 °C, exposure 20 min. Carrier gas—helium 99.9999%, injection mode—splitless, flow rate 1 mL/min. Ion source temperature—230 °C. Interface temperature 240 °C. Total ion current (TIC) monitoring mode was used. To analyze the obtained mass spectra, the NIST 2014 mass spectra library with automated mass spectral deconvolution and identification system (AMDIS version 2.72) was used.

### 2.7 Statistical analysis

Statistical analysis was performed separately for cellular and metabolomic data, as described below.

#### 2.7.1 Cellular assays (*TLR2*, *TLR4*, and *IL8* gene expression; IL-8 secretion)

Data were analyzed using Welch’s one-way ANOVA followed by Games-Howell multiple comparison tests. The Games-Howell procedure controls the family-wise error rate for multiple comparisons; therefore, no additional correction (e.g., Bonferroni) was applied. Analyses were carried out using R (version 4.2.2) on the BioRender platform. Results were considered statistically significant at *p* < 0.05.

#### 2.7.2 Metabolomic data (HS-GC/MS)

Peak areas calculated by AMDIS for the selected compounds were converted into relative abundances. Volatile compound percentages were estimated by summing the percentages of confidently identified compounds for each sample in the AMDIS database and recalculating them as a percentage of the total number of confidently identified compounds. This normalization was necessary to avoid errors due to unreliable matrix signals caused by noise. GC–MS data were processed using MetaboAnalyst 5.0 and GraphPad Prism 8.0.1. Values for each sample were considered paired and consistent, as confirmed by the ROUT outlier test (*Q* = 1%). Because normality of distribution could not be assumed for all sample groups, the non-parametric Mann–Whitney test was used for primary comparisons between groups. After natural log transformation, normalized data were analyzed using standard *t*-tests and ANOVA. Statistical significance was determined by a two-tailed *p*-value <0.05. Unsupervised principal component analysis (PCA) was used for dimensionality reduction; standard pre-processing procedures were applied. A chi-squared test was used to determine the method for imputing missing values; the missing-at-random (MAR) criterion was met, and the BPCA method was selected as the most appropriate. Normalization and scaling were then performed. Correlations between metabolites and gut microbiota composition were analyzed using the R “psych” package based on Spearman’s rank correlation coefficient.

## 3 Results

### 3.1 Effect of conditioned media from *Bacteroides* and *Phocaeicola* strains on *TLR2/TLR4* gene expression in HT-29 cells

**Figure 1:**
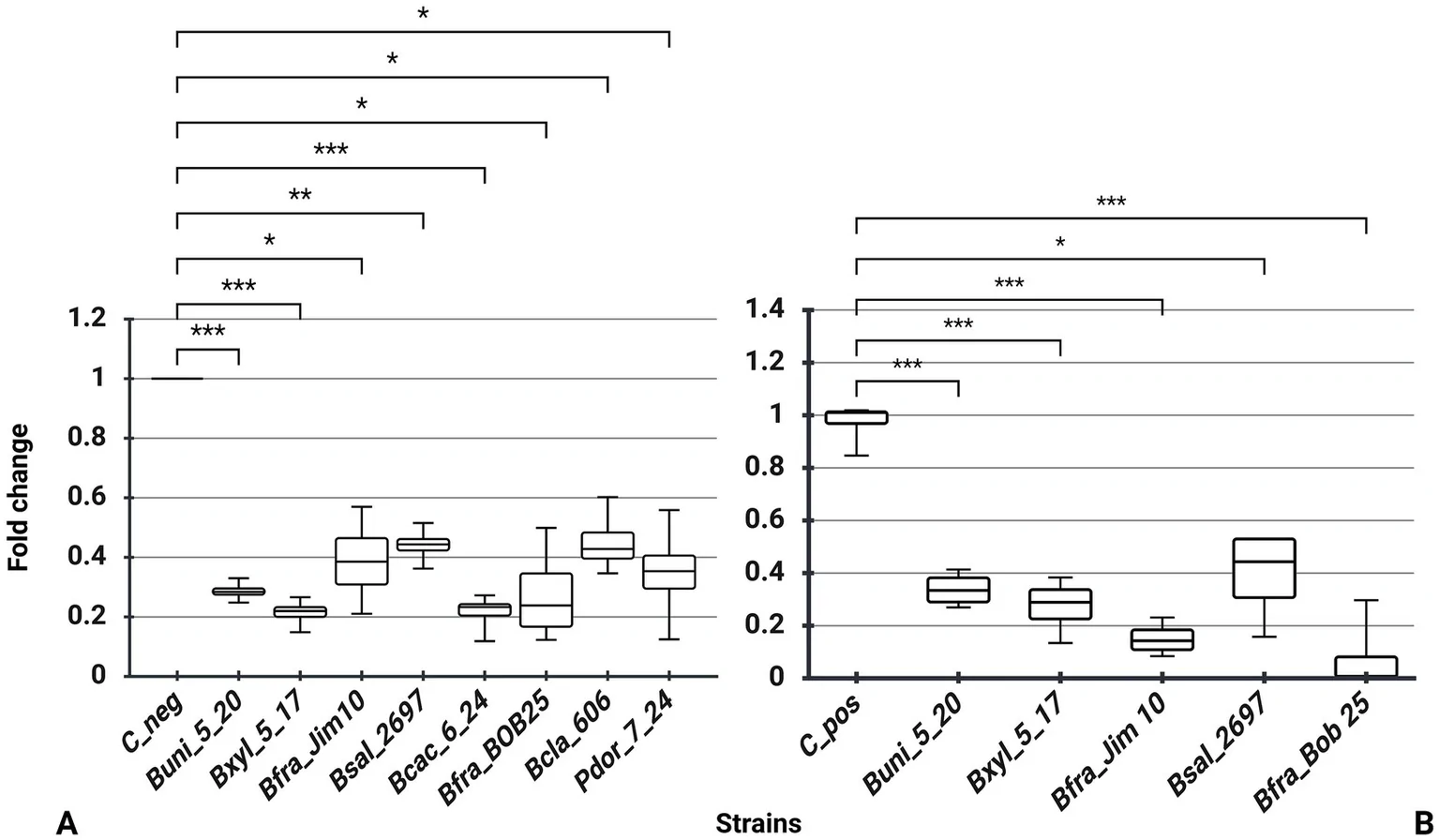
Effect of conditioned media from *Bacteroides and Phocaeicola str*ains on *TLR2* gene expression in HT-29 cells, without **(A)** and with **(B)** LPS (*E. coli* O55:B5) stimulation: C_neg—negative control (HT-29 cells without LPS treatment); C_pos—positive control (HT-29 cells treated with LPS); ***: *p* < 0.001, **: 0.001 < *p* < 0.01, *: *p* < 0.05 compared to C_neg and C_pos.

Analysis of the impact of conditioned media (CM) from the tested strains on *TLR2* gene expression revealed that CM from seven *Bacteroides* strains (*B. uniformis* EBA 5–20, *B. xylanisolvens* EBA 5–17, *B. fragilis* JIM10, *B. salyersiae* 2697, *B. caccae* EBA 6–24, *B. fragilis* BOB25, *B. clarus* 606) and one *Phocaeicola* strain (*P. dorei* EBA 7–24) significantly reduced *TLR2* expression compared to the negative control (C_neg). Moreover, five *Bacteroides* strains (*B. uniformis* EBA 5–20, *B. xylanisolvens* EBA 5–17, *B. fragilis* JIM10, *B. salyersiae* 2697, *B. fragilis* BOB25) exhibited a sustained ability to maintain low *TLR2* expression even under LPS stimulation of the HT-29 cell line (Figure 1).

**Figure 2:**
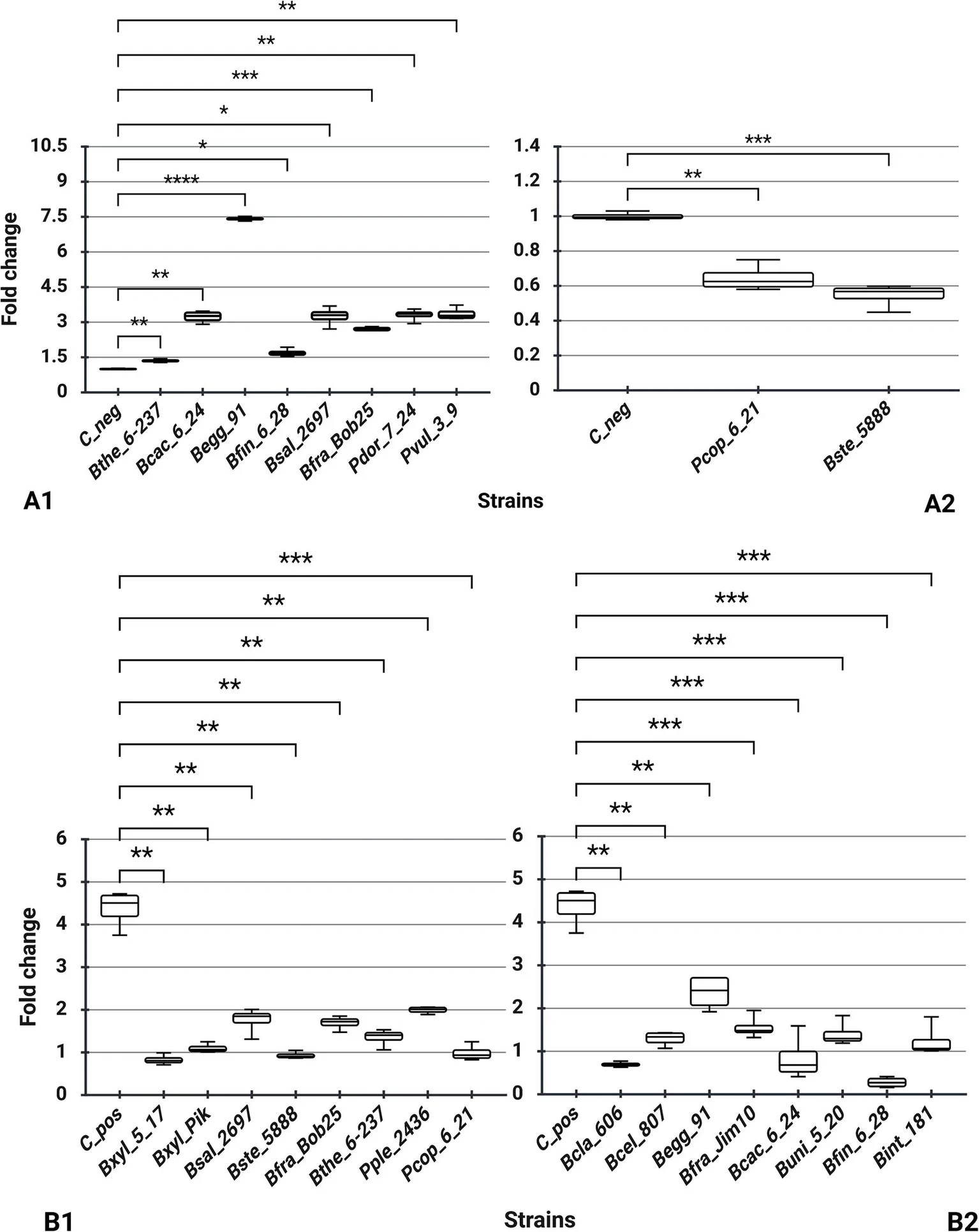
Effect of conditioned media from *Bacteroides and Phocaeicola str*ains on *TLR4* gene expression in HT-29 cells, without (**A1**,**A2**) and with (**B1**,**B2**) LPS (*E. coli* O55:B5) stimulation: C_neg—negative control (HT-29 cells without LPS treatment); C_pos—positive control (HT-29 cells treated with LPS); ***: *p* < 0.001, **: 0.001 < *p* < 0.01, *: *p* < 0.05 compared to C_neg and C_pos.

When examining the impact of CM on *TLR4* gene expression in HT-29 cells, it was found that CM from six *Bacteroides* strains (*B. thetaiotaomicron* 6–237, *B. caccae* EBA 6–24, *B. eggerthii* 91, *B. finegoldii* EBA 6–28, *B. salyersiae* 2,697, *B. fragilis* BOB25) and two *Phocaeicola* strains (*P. dorei* EBA 7–24, *P. vulgatus* EBA 3–9) significantly upregulated *TLR4* expression compared to untreated HT-29 control cells (C_neg). Conversely, CM from two strains (*P. coprocola* EBA 6–21 and *B. stercoris* 5888) significantly reduced *TLR4* expression, suggesting their potential ability to suppress TLR4–mediated inflammatory responses (Figures 2A1,A2). Further analysis of CM effects on LPS-induced *TLR-4* expression (*E. coli* O55:B5) demonstrated that 14 *Bacteroides* strains and 2 *Phocaeicola* strains significantly reduced *TLR4* expression in HT-29 cells relative to the positive control (C_pos) (Figures 2B1,B2). Among the tested strains, CM from *B. stercoris* 5888 and *P. coprocola* EBA 6–21 exhibited a pronounced suppressive effect on *TLR4* expression both under basal conditions and upon LPS induction. These findings highlight the diverse immunomodulatory properties of *Bacteroides* and *Phocaeicola* species and demonstrate the ability of the studied strains to regulate TLR2– and TLR4–mediated inflammatory processes, emphasizing their potential biomedical relevance and prospective applications in the prevention and treatment of diseases through the inhibition of TLR2 and TLR4 activation.

### 3.2 Effect of conditioned media from *Bacteroides* and *Phocaeicola* strains on *IL8* gene expression and secretion in HT-29 cells

**Figure 3:**
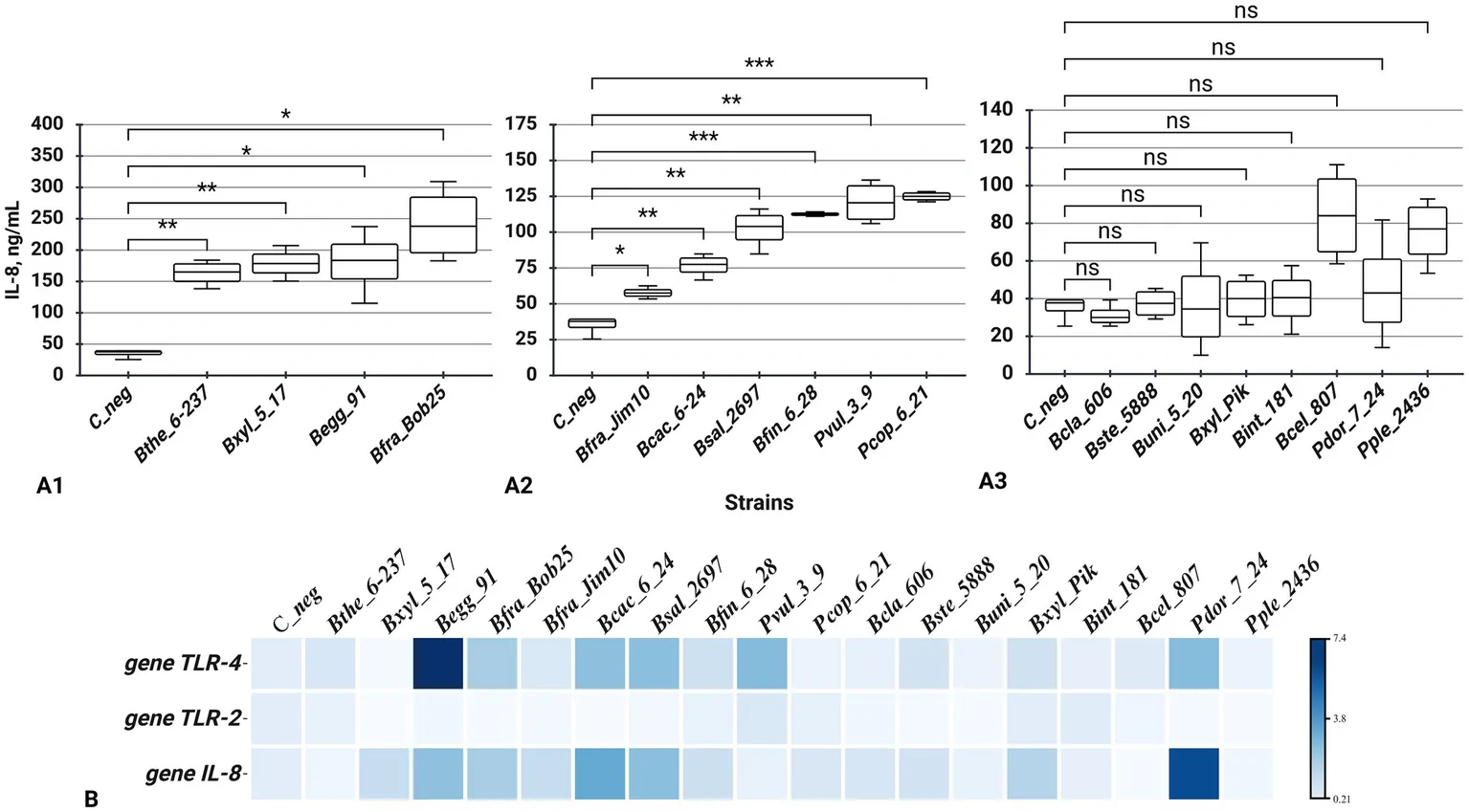
Effect of conditioned media from *Bacteroides* and *Phocaeicola* strains on IL-8 secretion in HT-29 cells without LPS (*E. coli* O55:B5) stimulation (**A1**–**A3**): C_neg—Negative control (HT-29 cells without LPS treatment); ***: *p* < 0.001, **: 0.001 < *p* < 0.01, *: *p* < 0.05 compared to C_neg. The heatmap (**B**) illustrates the relative expression levels of *IL8*, *TLR2*, and *TLR4* genes in HT-29 treated with conditioned media.

Following the assessment of conditioned media (CM) from *Bacteroides* and *Phocaeicola* strains on *TLR2* and *TLR4* genes expression, we further investigated their impact on *IL8* gene expression and chemokine secretion in the HT-29 cell line. As expected, HT-29 cells treated with LPS (C_pos) displayed high *IL8* gene expression and secretion compared to untreated cells (C_neg). Among the CM from 14 *Bacteroides* strains and 4 *Phocaeicola* strains, none induced a response comparable to *E. coli* O55:B5 LPS. All tested strains exhibited species- and strain-specific immunological activity, influencing *IL8* expression and secretion in HT-29 cells in a highly variable manner. Notably, CM from only two *Bacteroides* strains showed a consistent and significant effect on *IL8* gene expression under both CM-only treatment and CM combined with LPS, demonstrating either a pro-inflammatory (*B. caccae* EBA 6–24; *p* = 0.003 for CM, *p* = 0.04 for CM/LPS) or anti-inflammatory (*B. cellulosilyticus* 807; *p* < 0.0001 for CM, *p* = 0.0007 for CM/LPS) potential. Based on their effect on IL-8 secretion, the strains were divided into three groups: High pro-inflammatory potential: *B. thetaiotaomicron* 6–237, *B. xylanisolvens* EBA 5–17, *B. eggerthii* 91, *B. fragilis* BOB25, inducing IL-8 secretion in the range of 163.1 ± 20.8 to 242.0 ± 60.4 pg./mL (Figure 3A1). Moderate pro-inflammatory potential: *B. fragilis* JIM10, *B. caccae* EBA 6–24, *B. salyersiae* 2697, *B. finegoldii* EBA 6–28, *P. vulgatus* EBA 3–9, *P. coprocola* EBA 6–21, with IL-8 secretion ranging from 57.8 ± 3.9 to 124.9 ± 3.3 pg./mL (Figure 3A2). Minimal or negligible effect: *B. clarus* 606, *B. stercoris* 5888, *B. uniformis* EBA 5–20, *B. xylanisolvens* Pik, *B. intestinalis* 181, *B. cellulosilyticus* 807, *P. dorei* EBA 7–24, *P. plebeius* 2436, maintaining IL-8 secretion within 31.2 ± 6.1 to 84.4 ± 25.6 pg./mL (Figure 3A3), comparable to the negative control (C_neg: 35.1 ± 6.6 pg./mL).

**Figure 4:**
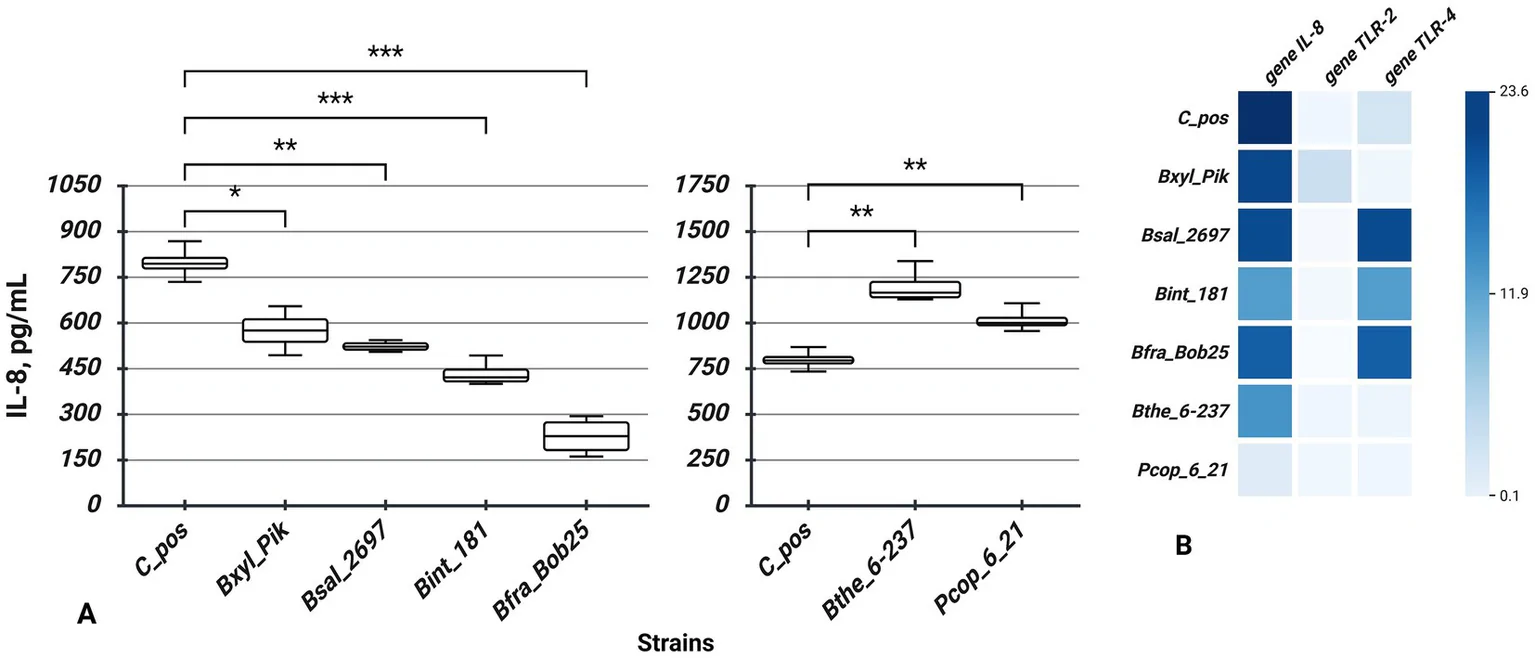
Effect of conditioned media from *Bacteroides* and *Phocaeicola* strains on IL-8 secretion in HT-29 cells stimulated with LPS (*E. coli* O55:B5) **(A)** C_pos—Positive control (HT-29 cells treated with LPS); ***: *p* < 0.001, **: 0.001 < *p* < 0.01, *: *p* < 0.05 compared to C_pos. The heatmap **(B)** illustrates the relative expression levels of *IL8*, *TLR2*, and *TLR4* genes in HT-29 cells stimulated with *E. coli* O55:B5 LPS and exposed to conditioned media.

Analysis of the effects of conditioned media on HT-29 cells stimulated with *E. coli* O55:B5 LPS revealed that four *Bacteroides* strains (*B. xylanisolvens* Pik, *B. salyersiae* 2697, *B. intestinalis* 181, *B. fragilis* BOB25) exerted a pronounced anti-inflammatory effect, reducing IL-8 secretion. In contrast, one *Bacteroides* strain (*B. thetaiotaomicron* 6–237) and one *Phocaeicola* strain (*P. coprocola* EBA 6–21) enhanced the pro-inflammatory activity of LPS. Notably, only CM from *B. intestinalis* 181 significantly reduced both *IL8* gene expression (*p* = 0.0003) and IL-8 protein secretion (*p* = 0.0004). Conversely, for *B. thetaiotaomicron* 6–237 (*p* = 0.001) and *P. coprocola* EBA 6–21 (*p* = 0.0005), the observed reduction in *IL8* gene expression was associated with potentiation of LPS-induced pro-inflammatory effects and an increase in IL-8 secretion (Figure 4).

### 3.3 Analysis of the volatile metabolites in culture supernatants and conditioned media

**Figure 5:**
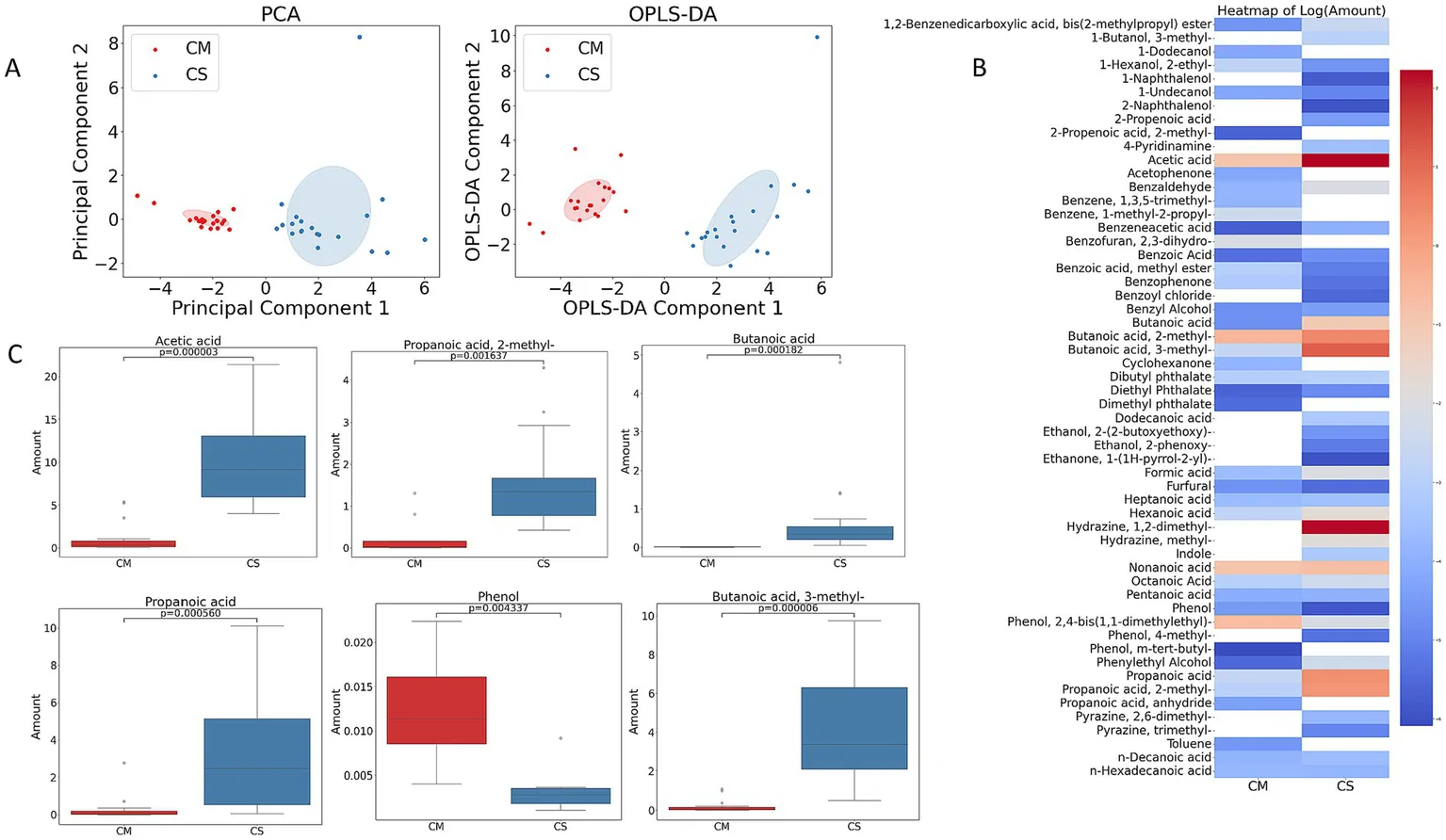
Total HS-GC/MS data obtained for CS and CM. **(A)** PCA and OPLS-DA data represent two independent groups of samples (CS and CM) according to the relative concentration of all detected volatile compounds. **(B)** Comparison of VOC composition for two independent groups of samples (CS and CM). Relative concentrations in the vapor phase were used. **(C)** The nonparametric Mann–Whitney test was used for the primary comparisons between groups. Statistical significance was determined by a two-sided *p*-value of less than 0.05. FDR correction was also applied. Box plots show the quantitative differences in the relative contents of metabolites detected in the analyzed groups (CS and CM).

A comparative metabolomic analysis was performed between bacterial culture supernatants (CS) and conditioned media (CM). Principal component analysis (PCA) demonstrated a clear separation between the CS and CM groups (Figure 5A). A heatmap revealed a higher abundance of volatile metabolites in CS compared to CM, particularly short-chain fatty acids (acetate, propionate, butyrate, and its branched derivatives) (Figure 5B). 2-Propenoic acid was exclusively present in CS, whereas 2-methyl-2-propenoic acid was detected specifically in CM. Ethanol and its derivatives, as well as indole and tryptophan, were found only in CS and were absent from CM. Conversely, 2,4-di-tert-butylphenol (2,4-DTBP) was detected exclusively in CM. Overall, all analyzed short-chain fatty acids were more abundant in CS than in CM.

**Figure 6:**
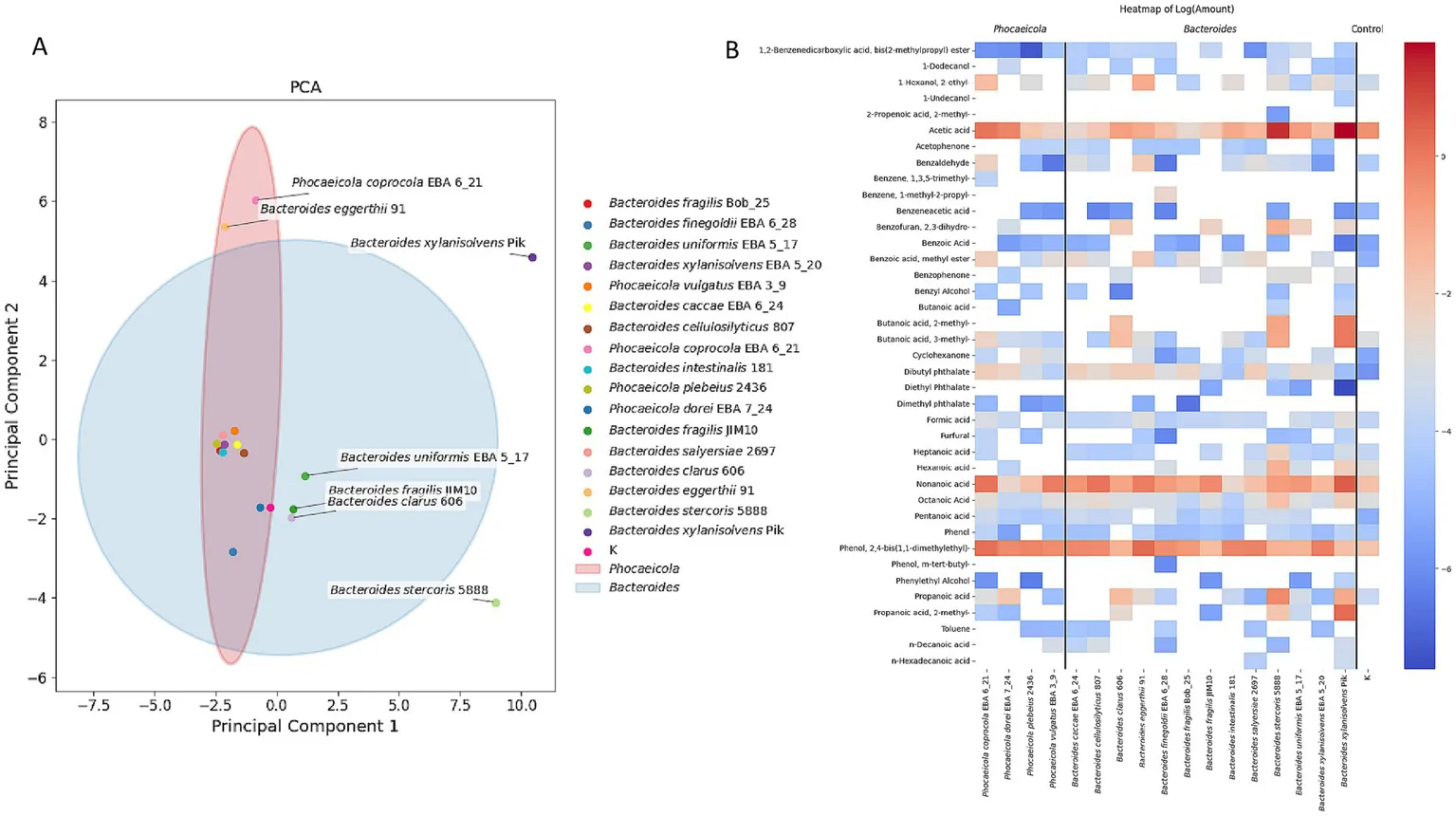
Total HS-GC/MS data obtained for CM. **(A)** PCA data represent two independent groups of samples (*Phocaeicola* CM metabolites and *Bacteroides* CM metabolites) according to the relative concentration of all detected volatile compounds. **(B)** Comparison of VOC composition for two independent groups of samples (*Phocaeicola* CM metabolites and *Bacteroides* CM metabolites). Relative concentrations in the vapor phase were used.

When comparing CM samples from different bacterial strains, PCA revealed differences in metabolic profiles. The strains *B. fragilis* JIM10, *B. clarus* 606, *B. uniformis* EBA 5–17, *B. stercoris* 5,888, and *B. xylanisolvens* Pik formed distinct clusters on the PCA plot (Figure 6). The metabolic profiles of *Phocaeicola* species were similar to those of *Bacteroides*. The heatmap showed that most strains produced nonanoic acid, 2,4-DTBP, and acetate. Propionate was detected in CM only for some strains (*P. dorei* EBA 7–24; *B. clarus* 606; *B. eggerthii* 91; *B. stercoris* 5888 and *B. xylanisolvens* Pik). The broadest spectrum of metabolites was observed for *B. stercoris* 5888 and *B. xylanisolvens* Pik.

**Figure 7:**
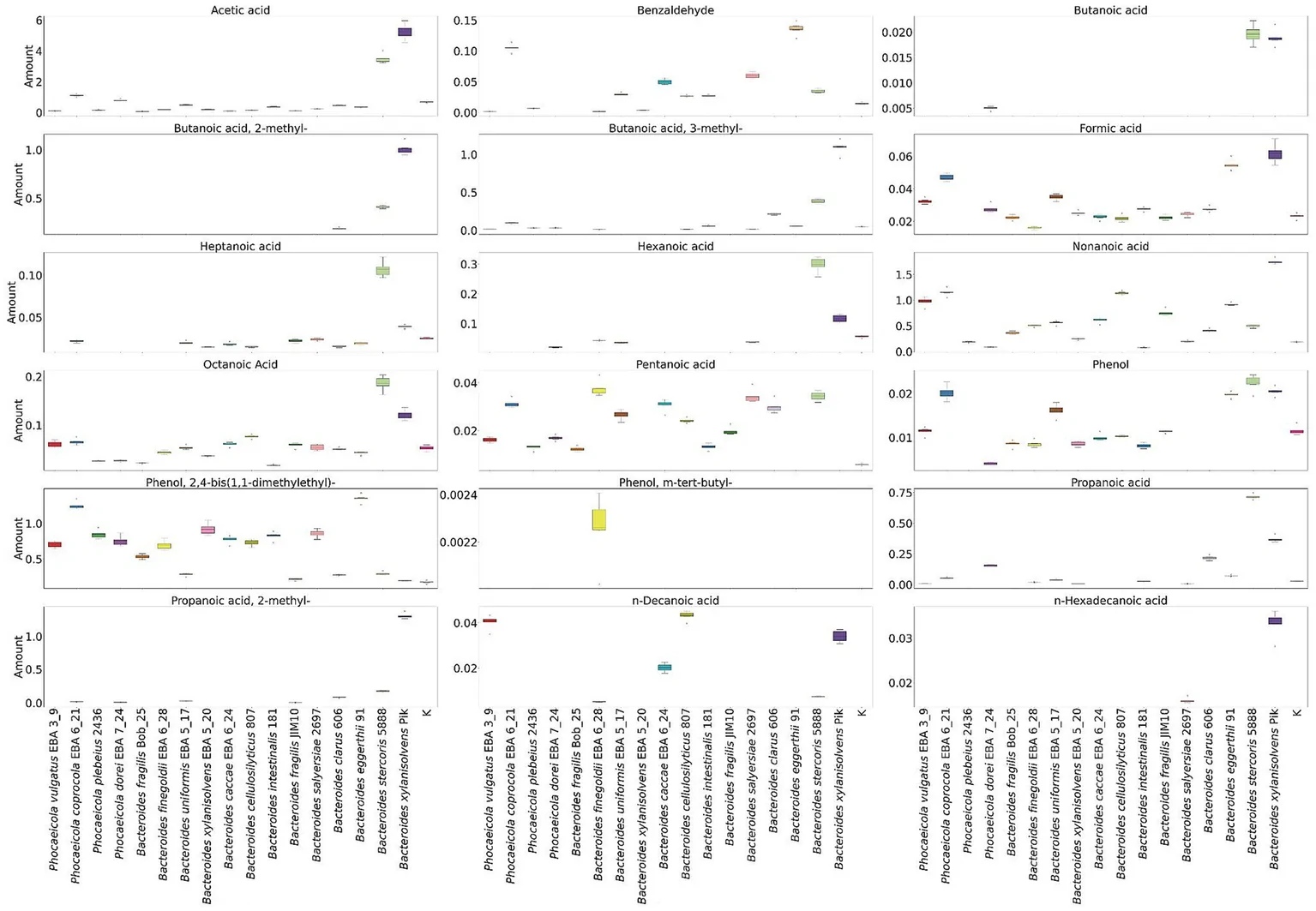
Relative abundances of volatile metabolites secreted by the majority of *Bacteroides* and *Phocaeicola* strains. The nonparametric Mann–Whitney test was used for the primary comparisons between groups. Statistical significance was determined by a two-sided *p*-value of less than 0.05. FDR correction was also applied. Box plots show the quantitative differences in the relative contents of metabolites detected in the analyzed groups (CS and CM).

Statistical analysis identified compounds secreted by the majority of the studied species (Figure 7). Acetate concentration was higher in *B. stercoris* 5888 and *B. xylanisolvens* Pik compared to the other strains. A similar pattern was observed for 2-methylbutanoic acid. Octanoic acid and acetate derivatives also showed higher levels in these two strains. 2,4-DTBP concentration was increased in all strains compared to the control. Pentanoic acid was increased in all tested strains. Additionally, phenol, nonanoic acid, and formic acid were widely detected among the studied bacteria.

## 4 Discussion

The present study evaluated the immunomodulatory potential of 18 strains of the genera *Bacteroides* and *Phocaeicola* by integrating volatile metabolite profiling (HS-GC/MS) with the analysis of TLR2/TLR4 expression and IL-8 secretion in HT-29 cells exposed to bacterial conditioned media (CM) (Supplementary Table S1). This design provides an *in vitro* approximation of metabolite-mediated effects, although contributions of other secreted or unstable bacterial components cannot be fully excluded. Our data demonstrate species- and strain-dependent variability in immunomodulatory activity, highlighting that even closely related species can exert divergent effects on the host immune system.

Consistent with previous reports, most short-chain fatty acids (SCFAs) and medium-chain fatty acids (MCFAs) are associated with reduced Toll-like receptor signaling and decreased IL-8 production, although these effects are context-dependent and may vary depending on cell type, concentration, and metabolic environment (Lin et al., 2015; Liu et al., 2023; Wang et al., 2023). In contrast, phenolic compounds such as phenol activate TLR pathways and stimulate IL-8 secretion (Rahman et al., 2021), and a similar pro-inflammatory effect has been described for benzaldehyde (Lamb and Rahman, 2023). Polysaccharide A of *Bacteroides fragilis*, by contrast, exerts relatively consistent immunomodulatory effects (Ramakrishna et al., 2019).

The molecular basis of SCFA-mediated immunomodulation is complex and involves at least two distinct mechanisms: inhibition of histone deacetylases (HDACs) and signaling through specific G-protein coupled receptors, primarily GPR41 (FFAR3) and GPR43 (FFAR2), which are expressed in intestinal epithelial cells (Lin et al., 2015; Koh et al., 2016; Akhtar et al., 2022). However, in intestinal epithelial cells, the effects of propionate and butyrate on the production of chemokines such as IL-8 and MCP-1 have been shown to be mediated primarily through HDAC inhibition, rather than through GPR signaling (Lin et al., 2015). Thus, the immunomodulatory effects observed in our HT-29 model are consistent with the epigenetic reprogramming driven by SCFAs via HDAC inhibition.

To understand the basis for the observed functional differences, we compared the metabolic profiles of two types of cultures: culture supernatants (CS) obtained after growing *Bacteroides* and *Phocaeicola* strains in rich Schaedler broth for 24 h, and CM obtained by transferring the same bacterial strains into glucose-free DMEM for an additional 18–20 h. As expected, CS contained higher levels of SCFAs (acetate, propionate, butyrate), reflecting active fermentative metabolism of *Bacteroides* in the nutrient-rich environment (Pan and Imlay, 2001). In contrast, CM exhibited a distinct signature. Specifically, 2-propenoic acid (acrylate) was present only in CS, whereas 2-methyl-2-propenoic acid (methacrylate) was detected exclusively in nutrient-limited CM. The presence of methacrylate only in CM suggests that its production may suggest that its production represents a potential metabolic adaptation of *Bacteroides* to environmental stress, possibly involving the redirection of branched-chain amino acid catabolism (leucine or valine) under carbon limitation, although this remains speculative and requires experimental validation. Ethanol, indole, and tryptophan were found only in CS, consistent with their accumulation under nutrient-replete conditions; indole is known to serve as a bacterial signaling molecule that influences host immune responses (Kumar and Sperandio, 2019). In contrast, 2,4-di-tert-butylphenol (2,4-DTBP) was detected exclusively in CM, supporting its proposed function as a stress-associated or quorum-sensing metabolite (Shagaleeva et al., 2023; Mishra et al., 2020; Jha et al., 2024; Al-abdullatif et al., 2025).

Strain-specific metabolomic profiling also revealed that *B. stercoris* 5888 and *B. xylanisolvens* Pik exhibited the broadest spectrum of metabolites, including elevated levels of acetate, 2-methylbutanoic acid, and octanoic acid. This is consistent with the known ability of these species to degrade various polysaccharides (starch, xylan) and their possession of a more flexible enzymatic apparatus (Despres et al., 2016; Vera-Ponce de León et al., 2020; Xu et al., 2024).

Statistical analysis showed that the majority of strains secreted a similar set of compounds, including acetate, 2,4-DTBP, valeric acid (pentanoate), phenol, nonanoic acid, and formic acid. These metabolites may reflect a shared metabolic pattern under nutrient-limited conditions. For example, propionate was detected in the CM of only some strains (*P. dorei* EBA 7–24, *B. clarus* 606, *B. eggerthii* 91, *B. stercoris* 5888, *B. xylanisolvens* Pik), indicating metabolic specialization that might define their ecological niches.

Thus, despite the existence of a common “metabolic core,” the production of specific metabolites (e.g., propionate) and their quantitative ratios differed substantially between strains, which underpinned the diversity of their immunomodulatory effects.

**Figure 8:**
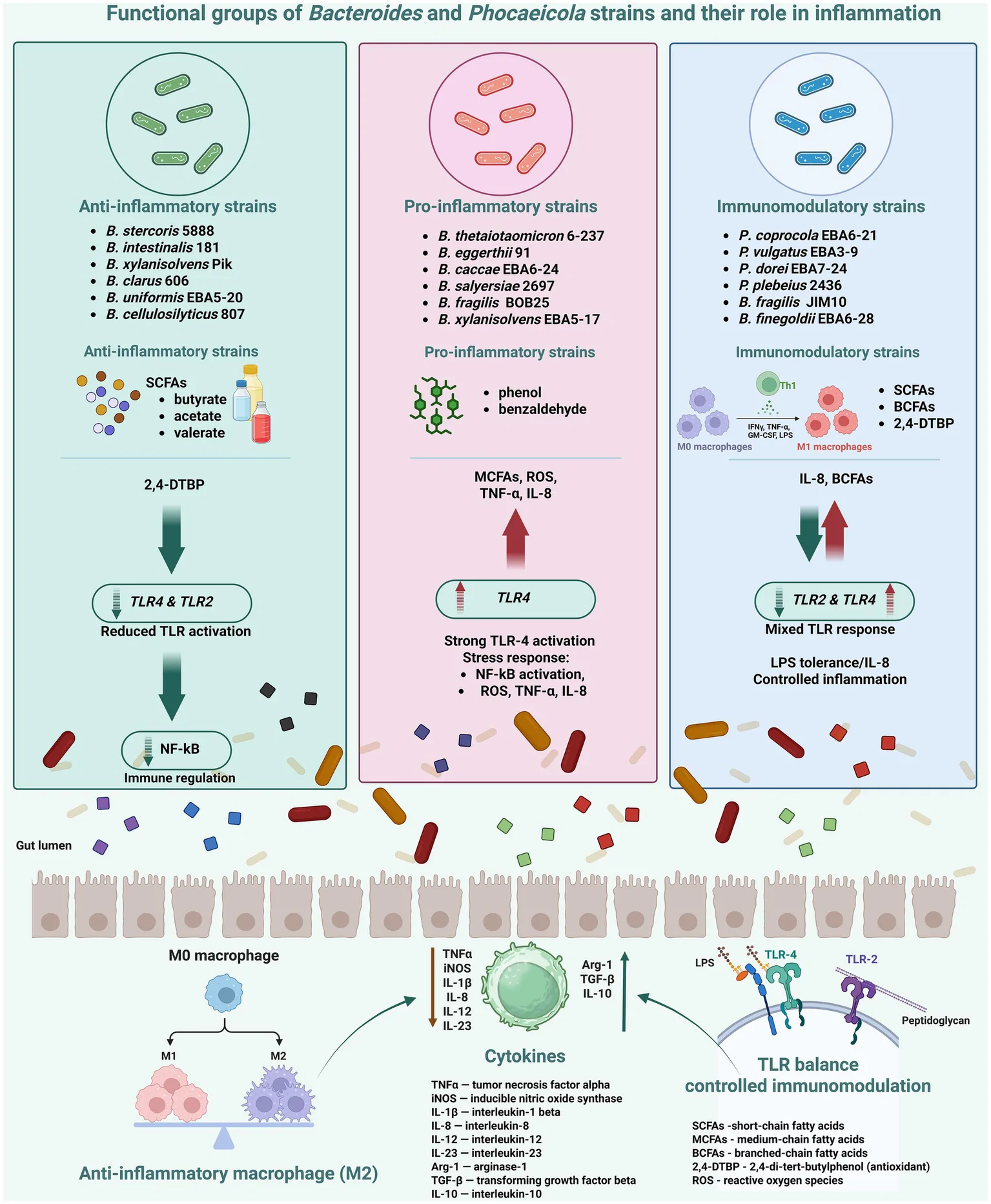
A comprehensive schematic summarizing all identified immunomodulatory effects of *Bacteroides* and *Phocaeicola inc*luding anti-inflammatory, mixed, and pro-inflammatory responses across all studied bacterial strains, based on metabolomic and secretome data, as well as the potential influence of these bacteria on the expression of Toll-like receptors 2 and 4, interleukin-8 gene expression, and interleukin-8 secretion.

Therefore, based on their properties and metabolic profiles, all tested bacteria can be tentatively classified into three response-associated groups based on *in vitro* immunomodulatory profiles: anti-inflammatory, pro-inflammatory, and those with mixed effects (Figure 8).

Strains that reduced *TLR4* activity and/or IL-8 secretion included *B. stercoris* 5888, *B. intestinalis* 181, *B. clarus* 606, *B. uniformis* EBA 5–20, *B. cellulosilyticus* 807, and *B. xylanisolvens* Pik. *B. stercoris* 5888 and *B. xylanisolvens* Pik demonstrated exceptionally high metabolic activity even under nutrient-limited conditions, indicating their resilience and suitability for further research. In addition to other metabolites such as acetate and propionate, these strains produced 2-methylbutanoic acid. This metabolite has been shown to alleviate murine experimental colitis by inducing immune tolerance. This effect was demonstrated for *Alistipes putredinis*, a bacterium belonging to the same order (*Bacteroidales*) as the strains analyzed in our study (Wu and Xu, 2024). Thus, the elevated production of 2-methylbutanoate by *B. stercoris* 5888 and *B. xylanisolvens* Pik may be associated with their anti-inflammatory-related activity under the tested conditions. Moreover, *Bacteroides intestinalis* 181 has previously been characterized as possessing moderate adhesive activity combined with a stable anti-inflammatory phenotype (Podoprigora et al., 2024), further supporting its probiotic potential.

Additionally, 3-methylbutanoic acid (isovaleric acid) was detected in the conditioned media of *B. stercoris* 5,888, *B. xylanisolvens* Pik, *B. clarus* 606, and *P. coprocola* EBA 6–21. This metabolite has previously been shown to possess anti-inflammatory activity, particularly through inhibition of NF-κB signaling and reinforcement of the epithelial barrier (Beaumont et al., 2026; Guo et al., 2025). Moreover, the anti-inflammatory potential of five-carbon fatty acids is further supported by studies on pentanoic acid (valeric acid), a structural analog of isovaleric acid. Valeric acid has been reported to suppress the production of the pro-inflammatory NF-κB subunit RELA (p65) while enhancing the expression of the anti-inflammatory cytokine IL-10 (Lin et al., 2023). Additionally, valeric acid acts as a selective inhibitor of class I histone deacetylases (HDACs), particularly HDAC3, thereby modulating inflammatory gene expression through epigenetic reprogramming (Yuille et al., 2018; Akhtar et al., 2022). Among the strains exhibiting pronounced anti-inflammatory activity, pentanoic acid producers included *B. cellulosilyticus* 807, *B. uniformis* EBA5-20, *B. intestinalis* 181, and *B. clarus* 606.

The pro-inflammatory group included strains that induced increased TLR4 expression and enhanced IL-8 secretion, despite the presence of short-chain fatty acids (SCFAs) in the conditioned media—metabolites generally associated with anti-inflammatory effects (Yu et al., 2025). For strains such as *B. caccae* EBA 6–24, *B. eggerthii* 91, and *B. salyersiae* 2,697, this pro-inflammatory phenotype may be linked to the presence of additional metabolites, such as benzaldehyde, which has been associated with intestinal inflammation and dysbiosis in clinical studies. Elevated benzaldehyde levels have been detected in fecal samples from patients with untreated celiac disease, correlating with a dysbiotic profile characterized by increased abundance of certain *Bacteroides* species and reduced levels of *Lactobacillus* and *Bifidobacterium* (Di Cagno et al., 2009). Similarly, higher benzaldehyde concentrations were observed in patients with non-celiac gluten sensitivity who experienced symptom worsening upon gluten reintroduction and were interpreted as a marker of inflammatory processes (Ferrocino et al., 2026). Although direct evidence for a causative role of benzaldehyde in TLR4-dependent IL-8 upregulation in intestinal epithelial cells is lacking, it is noteworthy that benzaldehyde has been shown to exert pro-inflammatory activity in other cell types. For instance, aerosol exposure to benzaldehyde significantly increased the production of the pro-inflammatory cytokine KC (the murine homolog of IL-8) in macrophages (Lamb and Rahman, 2023), suggesting that this compound possesses intrinsic immunostimulatory potential. In our experimental system, where HT-29 cells are exposed to a complex bacterial secretome, the presence of benzaldehyde could therefore contribute to the observed pro-inflammatory phenotype, potentially through synergistic interactions with other microbial components or by affecting the overall metabolic profile of the conditioned media. Thus, the pro-inflammatory activity of these strains is likely attributable to the combined action of multiple metabolites and bacterial components within the complex secretome, rather than to benzaldehyde alone. This underscores the need for further studies using purified compounds to dissect the specific contributions of individual metabolites to the observed immunomodulatory effects.

The pro-inflammatory role of *B. caccae* is supported by a causal link between its increased abundance and higher susceptibility to inflammatory bowel disease (Zheng et al., 2024). However, it is important to note that, like many other members of the genus *Bacteroides*, this species can also exhibit context-dependent anti-inflammatory properties, for instance, by suppressing LPS-inducible IL-8 secretion in HT-29 cells (Hiippala et al., 2020). These opposing observations highlight the strain- and context-specific nature of its immunomodulatory effects.

The pro-inflammatory potential of *B. eggerthii* is supported by *in vivo* evidence demonstrating that colonization with this species exacerbated DSS-induced colitis in mice (Dziarski et al., 2016). Furthermore, its abundance correlates with inflammatory markers in patients with carotid atherosclerosis (Chen et al., 2021) and ulcerative colitis (Le et al., 2025). Interestingly, despite these associations, the lipooligosaccharide (LOS) of the *B. eggerthii* strain 1_2_48FAA contains a hypo-acylated lipid A and acts only as a weak TLR4 agonist (Martín et al., 2025). The authors emphasize that the presence of galactofuranose (Galf) residues in the LOS raises important questions about its role in host immune interactions. Given that Galf serves as a ligand for the epithelial lectin ITLN-1, and the authors highlight the need to investigate the crosstalk between TLR4 and other receptors, such as lectins, Galf-ITLN-1 interaction may represent an alternative TLR4-independent mechanism of pro-inflammatory action for *B. eggerthii*. Collectively, these clinical and experimental findings are consistent with our *in vitro* observations and indicate that the immunomodulatory effect of *B. eggerthii* is determined by the balance between its pro-inflammatory and anti-inflammatory signals.

Similar patterns were noted for *B. salyersiae* 2697, *B. fragilis* BOB25, and *B. thetaiotaomicron* 6–237. Notably, these strains were characterized by relatively low overall metabolic and secretory activity, and several metabolites were present at reduced concentrations in the conditioned media. Nevertheless, experimental observations revealed increased IL-8 secretion and enhanced *TLR4* expression, consistent with a pro-inflammatory phenotype. This pattern is consistent with the known dual, context-dependent immunomodulatory properties of other *Bacteroides* species. For instance, *B. fragilis* and *B. thetaiotaomicron* have been shown to suppress the heightened inflammatory response induced by the viral mimic poly(I:C) in HT-29 cells, while simultaneously increasing basal IL-8 secretion (Pathmanathan et al., 2020). These findings suggest that *Bacteroides* spp. can exert both pro- and anti-inflammatory effects depending on the host cell activation state, which aligns with our observation that these strains enhance basal inflammation while potentially attenuating inflammatory responses in an LPS-induced context. Interestingly, *B. fragilis* BOB25 exhibited a context-dependent immunomodulatory profile: while it increased *TLR4* expression and IL-8 secretion in untreated HT-29 cells, it suppressed both *TLR4* expression and IL-8 secretion in LPS-stimulated cells. This pro-inflammatory activity in the basal state is consistent with the known ability of enterotoxigenic *B. fragilis* (ETBF) strains to activate IL-8 expression in HT-29 cells through Stat3-dependent signaling (Purcell et al., 2022).

The third group, representing bacteria with mixed immunomodulatory effects, displayed the most complex and heterogeneous responses. In these cases, the effects that could be predicted from the metabolic composition did not consistently align with the observed cellular responses, suggesting the presence of additional regulatory factors. This group included *P. vulgatus* EBA 3–9, *P. dorei* EBA 7–24, *P. plebeius* 2436, *P. coprocola* EBA 6–21, *B. fragilis* JIM10 and *B. finegoldii* EBA 6–28. Within this category, some strains demonstrated a predominance of pro-inflammatory effects, whereas others exhibited partial anti-inflammatory activity. For instance, *P. vulgatus* EBA 3–9 exhibited a predominantly pro-inflammatory profile, characterized by increased TLR4 expression and enhanced IL-8 secretion. The literature contains conflicting data regarding the pro-inflammatory potential of *P. vulgatus*. In the study by Cuív et al. (2017), two strains of *B. vulgatus*, ATCC 8482 and PC510, were investigated. Both strains activated NF-κB in a strain- and growth phase-dependent manner in HT-29 cells. Notably, strain ATCC 8482 induced nuclear translocation of NF-κB-p65 and upregulated the expression of pro-inflammatory genes, including IL-8, TNF, and CXCL-10. By contrast, Pathmanathan et al. (2020) demonstrated that under conditions of poly(I:C)-induced inflammation, *B. vulgatus* exerted an anti-inflammatory effect by suppressing the inflammatory response. Importantly, both of these observations were made using models in which epithelial cells were exposed to intact bacterial cells rather than to cell-free supernatants. Given that our conditioned media were obtained by centrifugation and filtration to remove bacterial cells, we cannot exclude the possibility that residual bacterial structural components, such as outer membrane vesicles or cell wall fragments, may have been retained in the conditioned media and thus contributed to the observed pro-inflammatory effects.

Additional evidence for the complexity of this immunomodulation has emerged at the molecular level. In a study employing functional metagenomics, Cohen et al. (2015) identified a specific metabolite, N-acyl-3-hydroxypalmitoyl-glycine, termed commendamide. This metabolite was detected in the culture supernatants of *B. vulgatus* ATCC 8482 and other members of the order *Bacteroidales*. Commendamide activates the G-protein-coupled receptor G2A/GPR132, which has been implicated in the regulation of inflammatory processes. Collectively, these observations highlight that even within a single bacterial species, different strains may employ distinct molecular mechanisms to modulate host immune responses, and that pro-inflammatory effects may arise from both bacterial structural components and secreted metabolites.

In contrast, *P. dorei* EBA 7–24 demonstrated a mixed anti-inflammatory pattern, with increased *TLR4* expression but no significant change in IL-8 secretion. Particularly notable was *P. plebeius* 2436, which exhibited an essentially neutral effect, with no detectable changes in either IL-8 secretion or *TLR4* gene expression. Although weak anti-inflammatory activity might have been expected based on metabolite composition, this effect was not clearly observed under our experimental conditions. Nevertheless, the absence of a pro-inflammatory response in our HT-29 model is consistent with the protective role attributed to *P. plebeius in vivo*. Chen et al. (2025) demonstrated that *P. plebeius* possesses anti-inflammatory and anti-tumorigenic properties in a mouse model of colitis-associated colon cancer (Chen et al., 2025). Thus, although the anti-inflammatory activity of *P. plebeius* 2436 was not overtly detectable in our *in vitro* system, the lack of a pro-inflammatory response aligns with the protective profile of this species, suggesting that its immunomodulatory effects may be context-dependent and require in vivo conditions.

The immunomodulatory activity of *Bacteroides* and *Phocaeicola* strains is governed by the balance between pro-inflammatory metabolites (e.g., benzaldehyde, phenols) and anti-inflammatory signals, such as HDAC-mediated epigenetic reprogramming driven by short-chain fatty acids and their structural analogs. Strains with a favorable metabolic profile and high secretory activity, such as *B. stercoris* 5888 and *B. xylanisolvens* Pik, hold promise as probiotic candidates, whereas strains with a predominantly pro-inflammatory secretome may contribute to chronic inflammation if they overgrow in the dysbiotic gut. Thus, the integration of metabolomic and functional immune profiling provides a rational basis for strain-specific selection, enabling the prediction of immunomodulatory outcomes and the development of targeted microbiome-based strategies for the correction of dysbiotic conditions.

### 4.1 Potential clinical applications and translational relevance

The strain-specific differences in metabolite production and immunomodulatory activity observed in this study may have important translational implications for the development of microbiome-based therapeutic strategies. Identification of bacterial strains capable of modulating host immune responses through the production of bioactive metabolites could facilitate the rational selection of next-generation probiotics or live biotherapeutic products for maintaining intestinal homeostasis and supporting the management of inflammation-associated disorders. Furthermore, characterization of strain-specific secretomes may contribute to the identification of metabolite-based biomarkers and provide a foundation for developing postbiotic formulations containing defined microbial metabolites or secreted components.

These findings provide a preliminary framework for understanding microbial symbiosis at the metabolic level and for exploring associations between strain-specific metabolite profiles and host immune responses. Our results may contribute to the identification of candidate symbiont strains that are potentially associated with maintenance of eubiosis or attenuation of dysbiosis.

However, the present findings are based on an in vitro epithelial cell model, and their clinical relevance requires further validation in additional cellular systems, animal models, and human studies before any therapeutic applications can be considered.

### 4.2 Limitations of the study

Several limitations of the present study should be acknowledged. First, all experiments were performed using a single intestinal epithelial cell line (HT-29). This choice was made to ensure experimental standardization and reproducibility across a large and diverse panel of bacterial strains and conditioned media, which was essential for comparative screening. While this approach minimizes inter-assay variability and allows robust strain-to-strain comparisons, it also limits the direct extrapolation of the findings to other intestinal epithelial models or immune-relevant cell systems. Future studies should therefore validate the observed effects in additional epithelial and immune cell models to strengthen biological generalizability.

Second, the evaluation of inflammatory responses was based on a restricted set of markers, including *TLR2*, *TLR4* expression, and IL-8 secretion. Although these parameters are widely accepted indicators of innate immune activation, they only partially reflect the complexity of host inflammatory signaling networks. A more comprehensive characterization using an expanded panel of cytokines, chemokines, and downstream signaling molecules would provide deeper mechanistic insight into strain-specific host responses.

Finally, the present study did not include higher-resolution functional assays such as flow cytometry or confocal microscopy. These approaches would enable more detailed assessment of cellular heterogeneity, receptor-level dynamics, and spatial organization of immune responses. Their inclusion in future work will be important for validating and extending the current findings at the mechanistic level.

## Data Availability

The raw data supporting the conclusions of this article are included in the Supplementary Material. The HS‑GC/MS datasets generated for this study have been deposited in the Zenodo repository under accession number https://doi.org/10.5281/zenodo.21702022 (Kardonsky, 2026). The datasets can also be obtained from the corresponding authors upon reasonable request.
